# Variation in volatile organic compounds in fruits of Iranian Capparis spinosa L. accessions

**DOI:** 10.1016/j.sjbs.2021.04.077

**Published:** 2021-05-01

**Authors:** Fereidon Alipour, Amrollah Nabigol, Esmail Nabizadeh

**Affiliations:** a Department of Horticulture, Abhar Branch, Islamic Azad University, Abhar, Iran; b Department of Agronomy, Mahabad Branch, Islamic Azad University, Mahabad, Iran

**Keywords:** *Capparis* spinosa, Essential oil content, Medicinal plant, Altitude

## Abstract

In the present study, essential oil (EO) content and phytochemical variation were evaluated in the fruits of 10 Iranian *Capparis spinosa* accessions. The accessions were collected from their natural habitats of Iran and grown together in field conditions. The EOs content ranged from 0.55 to 1.46%. The correlation analysis revealed a significant negative correlation between the EO content and altitude of the sampling locations (P < 0.01; r = −0.84). GC–MS analysis revealed 31 compounds in the EOs of the accessions, mainly being isopropyl isothiocyanate (5.5–13.7%), methyl sulfonyl heptyl isothiocyanate (4.6–15.6%), butyl isothiocyanate (3.6–10.6%), γ-terpinene (4.4–9.2%), and thymol (22.9–37.1%). According to the cluster analysis, the accessions were classified into three groups. Principal component analysis (PCA) demonstrated that the first and second PCs1 confirmed more than 76% of the total variation in the phytochemical components among the *C. spinosa* samples. Our results revealed that the sampling altitude was the most effective factor in explaining this variation.

## 1 Introduction

The genus *Capparis* belongs to the Capparidaceae family and contains 250 species in the world (Inocencio et al., 2006, Tlili et al., 2011) *Capparis spinosa* L. is a common perennial medicinal herb that grows in wild and cultivated conditions. Its local name is “Kabar” in Persian. It grows 0.3–1 m tall and has roots that can penetrate 6–10 m down. It is widely distributed in different countries, e.g. Morocco, Armenia and Iran (Rivera et al., 2003). *C. spinosa* has multiple pharmaceutical uses, antimicrobial, hepatoprotective, antioxidant, cardiovascular, anti-cancer and hypoglycemic properties (Moghadamnia et al., 2019, Mollica et al., 2019). The plant has nutritional values which are useful in traditional medicine for the treatment of many ailments. It has a significant role as a diuretic agent and as an astringent herb. The fruits and roots of *C. spinosa* are commonly used to treat hemorrhoids in Iran (Miraldi et al., 2001). The essential oils (EO) are extracted from different parts of *C. spinosa*, i.e. leaves, fruits and roots (Afsharypuor et al., 1998). Methyl, isopropyl and set-butyl isothiocyanates are the major volatile oils in fruits and roots of *C. spinosa*. Also, the EO content is higher in the fruits than in the roots and leaves (Afsharypuor et al., 1998). Esmaeilzadeh-Bahabadi and Najafi, (2016) reported that the EO of its fruits is rich in thymol. Meanwhile, further evaluations of the EO of this medicinal plant can benefit the current knowledge about its bioactive compositions, thereby generating a superior alternative to chemical, synthetic drugs.

The EO components can be affected by many factors such as plant genetics, soil conditions, altitude, growth phase, different plant parts and environmental factors (Hassanabadi et al., 2019). Therefore, exploring phytochemical variations among the accessions of each medicinal plant can lead to new discoveries about valuable components and their ratios (Ebrahimi et al., 2012). To the best of our knowledge, there is limited information on the variation of phytochemical components in EOs that are extracted from the fruits of Iranian *C. spinosa* accessions. The objective of the present study was to assess the phytochemical variation among EOs that were extracted from the fruits of Iranian *C. spinosa* accessions.

## 2 Material and methods

### 2.1 Plant material and isolation of EO

The seeds of 10 *C. spinosa* accessions were gathered from Iran. Information on coordinate and habitat characteristics of the sampling locations is presented in Table 1. To break the seed dormancy, the seeds were soaked in gibberellic acid (500 ppm) for 12 h (Tafti et al., 2012). The seeds were sown under greenhouse conditions in a medium containing peat moss and perlite (1:2 v/v). After establishment, the seedlings were transferred to filed conditions.

**Table 1:** Information of 10 *C. spinosa* accessions investigated in the present study.

Genotype	Origin	Altitude	Longitude	Latitude
G1	Maku, West Azarbaijan, Iran	1311	44°31′E	39°15′N
G2	Sardasht, West Azarbaijan, Iran	1522	45°47′E	36°15′N
G3	Marand, East Azarbaijan, Iran	1331	45°45′E	38°30′N
G4	Ahar, East Azarbaijan, Iran	1342	47°00′E	38°35′N
G5	Paveh, Kermanshah, Iran	1533	46°22′E	35°03′N
G6	Ravansar, Kermanshah, Iran	1346	46°40′E	34°43′N
G7	Baneh, Kurdistan, Iran	1522	45°53′E	35°59′N
G8	Marivan, Kurdistan, Iran	1302	46°25′E	35°30′N
G9	Abhar, Zanjan, Iran	1542	49°13′E	36°09′N
G10	Qeydar, Zanjan, Iran	1973	48°59′E	36°12′N

The filed site was located in Sardasht city at 1522 m asl, 36°15́N and 45°47́E, in West Azerbaijan Province. The average annual rainfall and temperature of the studied site were 850 mm and 13.3 °C, respectively.

At the maturity stage, the ripe fruits were collected. To extract the EOs, the fruits were dried at 25 °C. The powdered fruits (30 g) were hydro-distilled using a Clevenger-type apparatus for 2.5 h. The EO was calculated as the volume of EO per 30 g of dry weight.

### 2.2 Determination of EO components

The gas chromatography-mass spectrometry (GC–MS) was carried out on a Hewlett-Packard 6890 model, equipped with an HP-5MS capillary column (30 m × 0.25 mm i.d. × 25 µm film thickness). Helium and 70 eV were used as the carrier gas and ionization energy, respectively. To determine the EO compositions, retention index (RI) was calculated based on retention times for n-alkanes (C6-C24) and was compared with a mass finder in 2.1 Library, according to published data in the literature, and the internal reference MS Library (Wiley 7.0; Adams, 2007, König et al., 2001, Mclafferty and Stauffer, 2009).

### 2.3 Data analysis

Principal component analysis was performed using the SAS software. The Heat maps of the clusters, correlation analysis and of three plots were conducted using Metabo Analyst (Xia and Wishart, 2016).

## 3 Results

### 3.1 EO content

**Fig. 1:**
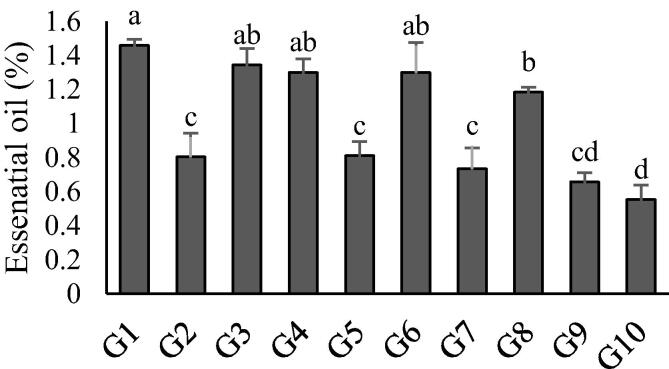
Variation of essential oil yield of 10 *C. spinosa* accession.

**Fig. 2:**
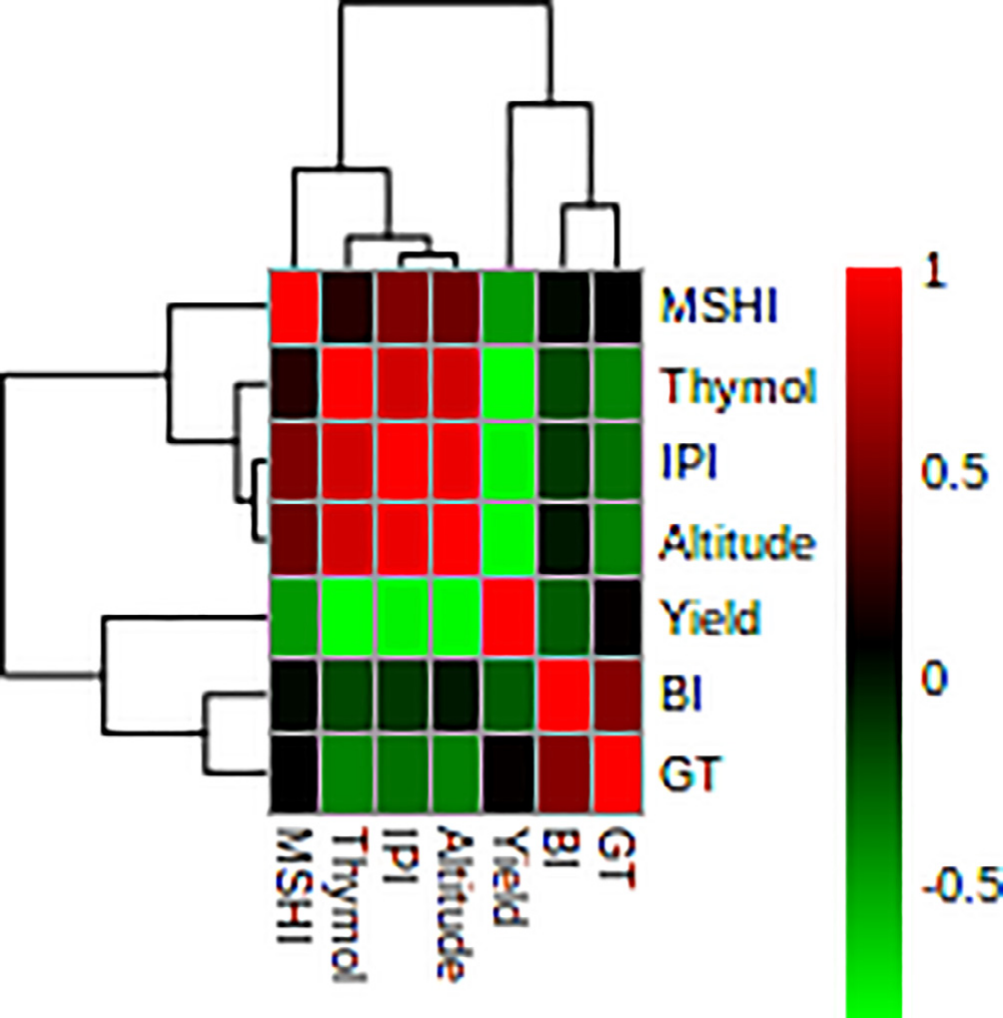
Heat map of the correlations among the top five components and yield of *C. spinosa*. MSHI: Methyl sulfonyl heptyl isothiocyanate; IPI: Iso propyl isothiocyanate, BI: Butyl isothiocyanate, GT: γ-terpinene.

According to the results of the analysis of variance, there were significant differences among EO contents in the accessions (P < 0.01; data not shown). The mean values of the EO contents are outlined in Fig. 1. Two accessions from Zanjan province (G8 and G10) had the lowest EO contents, compared to other accessions, although the highest EO content was recorded in the G1 accession (1.46%). The correlation analysis was applied to describe the relationship between EO content and altitude (Fig. 2). The result of the analysis indicated a significant negative correlation between them (P < 0.01; r = −0.84).

### 3.2 EO composition

**Fig. 3:**
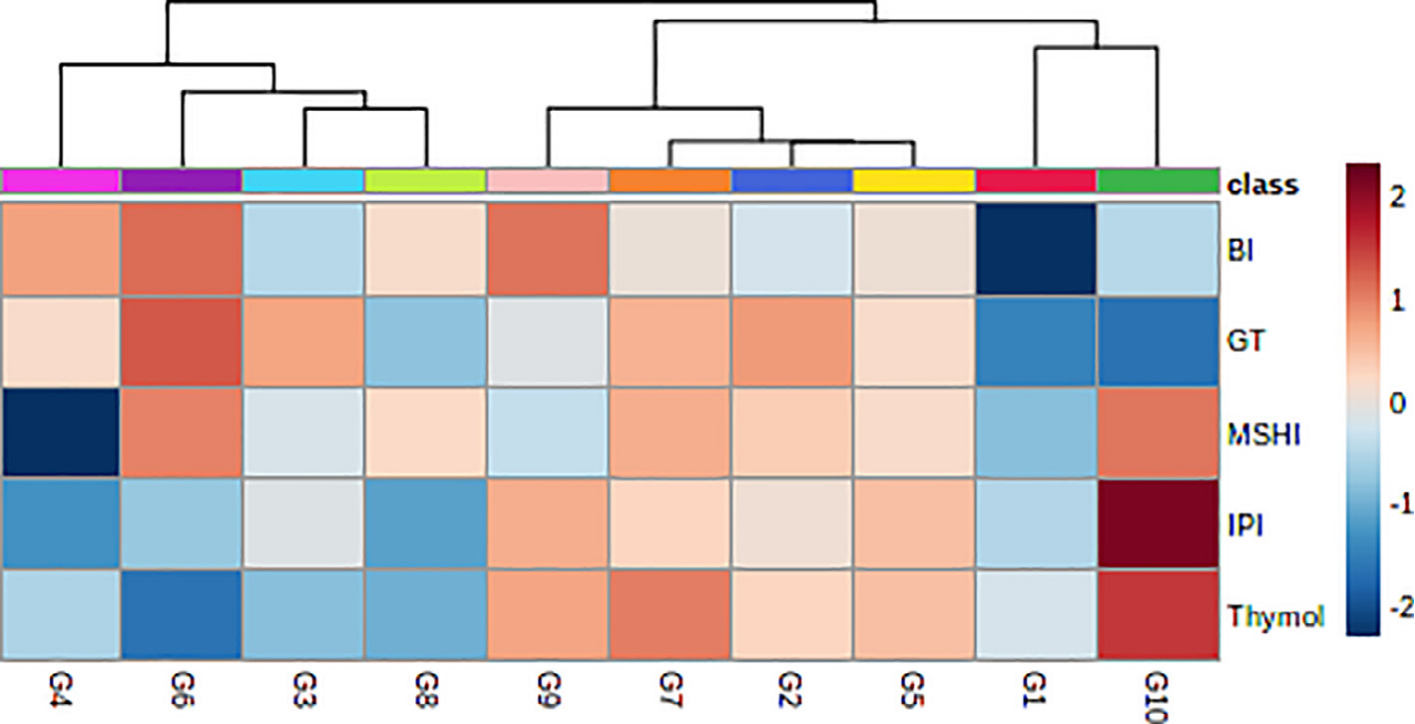
Heat map of the cluster analysis among the studied genotype based on the top five components using the ward's method.

**Fig. 4:**
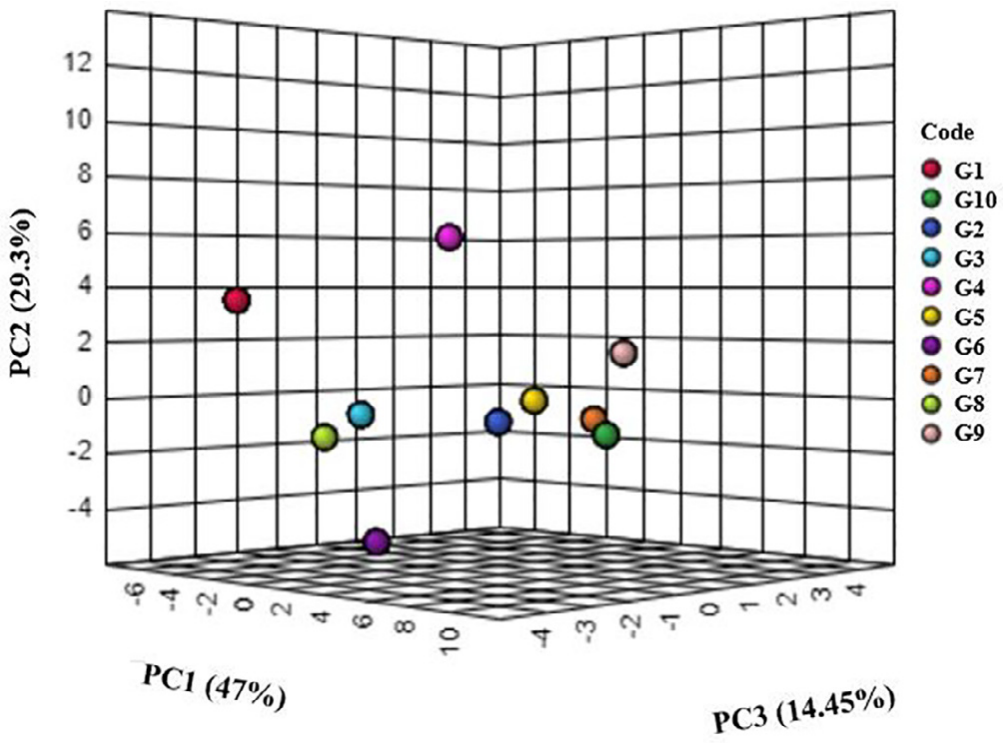
Three plots derived from the principal component analysis.

The EO composition was described using GC–MS analysis. The GC–MS revealed 31 components in the Iranian *C. spinosa* accessions (Table S1). Many of the detected components were observed in all of the accessions. The chief constituents of the EOs in the Iranian *C. spinosa* samples were iso propyl isothiocyanate (5.5–13.7%), methyl sulfonyl heptyl isothiocyanate (4.6–15.6%), butyl isothiocyanate (3.6–10.6%), γ-terpinene (4.4–9.2%), and thymol (22.9–37.1%). There is limited information on the variation of phytochemical components of EOs that are extracted from *C. spinosa*. According to the results of the present study, the accessions were rich in thymol. To classify the accessions, cluster analysis was applied by the top five major compositions. Based on the analysis, *C. spinosa* accessions were classified into three groups (Fig. 3). The first cluster contained two samples (G1 and G10). The G10 accession had the highest amounts of isopropyl isothiocyanate (13.7%), methyl sulfonyl heptyl isothiocyanate (15.6%) and thymol (37.1%). This accession was collected from the highest altitude (1973 m), compared to the other accessions. Meanwhile, accessions in the second and third groups were collected from altitudes of 1522–1542 m and 1302–1342 m, respectively. The PCA was applied according to the five main compositions (Table 2). The results of PCA revealed that the first principal components (PC1) confirmed about 47% of total variation among the accessions. This variation could be due to the altitude of the sample collection area. The PC1 correlated positively with isopropyl isothiocyanate and thymol components, while these components correlated positively and significantly with each other (P < 0.01; Fig. 2). In addition, γ-terpinene had a negative correlation with the PC1. Methylsulfonyl heptyl isothiocyanate and butyl isothiocyanate had a positive correlation with PC2, which explained 29% of the variance among the accessions. The PCA analysis plot separated the G1 accession from the other accessions, a result that corresponds closely with the cluster analysis (Fig. 4). A slight difference between cluster analysis and PCA plots occurred because only three components were used and, thus, explained not all but the major portion (90%) of the variation.

**Table 2:** PCA based on the five top EO compounds of 10 *C. spinosa* accessions.

Label	Major compound	Principal components	
		PC1	PC2
1	Iso propyl isothiocyanate	0.9	0.34
2	Thymol	0.87	0.12
3	γ-terpinene	−0.6	0.64
4	Methyls sulfonyl heptyl isothiocyanate	0.45	0.64
5	Butyl isothiocyanate	−0.43	0.7
–	Eigenvalue	2.34	1.47
–	% of variance	0.47	0.29
–	Cumulative%	0.47	0.76

## 4 Discussion

According to the results of the present study, a high EO variation was observed in the accessions. Afsharypour et al (1998) obtained 0.08, 0.9, and 0.02% EO contents in the dried leaves, ripe fruits, and roots of *C. spinosa*, respectively. According to the literature review, the highest EO content was obtained from the *C. spinosa* fruits, compared to the other plant parts. Afsharypour et al (1998) merely analyzed one genotype and obtained 0.9% EO from the fruit. In the present study, however, 10 accessions were used, the EOs of which ranged from 0.55 to 1.46%. This range shows a high level of EO diversity among the Iranian *C. spinosa* samples. It could be due to many genetic and environmental factors (Farajpour et al., 2017). Based on the results, the lower the altitude, the higher the temperature, and so it seems that plants of lower altitudes are usually exposed to higher temperatures. This stress (high temperature) can be expected to cause an increase in the EO content of plants that originate in lower altitudes. Haider et al., 2009, Shams et al., 2016 reported that a decrease in altitude corresponds with an increase in the EO contents of *Artemisia roxburghiana* and *Coriandrum sativum*. These reports are in good agreement with our results, although opposite results have been reported in the case of *Ferula assa-foetida* (Hassanabadi et al., 2019).

In this study, 31 components were detected in the Iranian *C. spinosa* accessions. Many of these components were observed in all accessions. Esmaeilzadeh-Bahabadi and Najafi, (2016) reported 33 components in the EO of *C. spinosa* fruits, whereas Afsharypour et al. (1998) obtained six compositions only. According to a previous report, after extracting EO from the ripe fruits of this species, the main compositions of the EO were methyl, isopropyl and set-butyl isothiocyanates (Afsharypuor et al., 1998).

Based on the results of this study, the 31 accessions were rich in thymol, a component that has a wide range of applications in medical, agricultural and food industries. Thymol is classified as a safe molecule by the U.S Food and Drug Administration (da Silva Mendes et al., 2011, Gill et al., 2016, Gruľová et al., 2020, Kollanoor Johny et al., 2010, Kumari et al., 2018). Therefore, it seems that *C. spinosa* could be considered as a top medicinal plant because of its rich thymol content and other components (Fig. 1). The quality and quantity of the EO, as well as the chemical compositions of the EO in medicinal plants, are affected by many factors such as genetic variables, climate, physiological properties, soil conditions and geographical origin (Farajpour et al., 2017). In the present study, the accessions were classified into three groups, based on the altitude of the sampling locations. This shows that the altitude not only affects the EO content but also can change their chemical compositions and ratio. There have been several reports on different medicinal plants in which the EO content and their chemical components are affected by altitude. Examples of such medicinal plants are *Ferula assa-foetida* (Hassanabadi et al., 2019; Nasiri, Bezenjani et al., 2017) and *A. aucheri* (FarhangSardrodi et al., 2017). According to the results of the present study, high levels of diversity were observed in EO contents and in their composition, while most of this diversity can be explained by altitude.

## 5 Conclusion

In the present study, the EO content and chemical components of 10 Iranian *C. spinosa* accessions were evaluated. The results showed high levels of phytochemical diversity among the accessions, most of which turned out to be rich in thymol. In addition, the results revealed that the altitude of the sampling locations is the most effective factor in causing these variations among the EOs and their phytochemical components.

## Declaration of Competing Interest

The authors declare that they have no known competing financial interests or personal relationships that could have appeared to influence the work reported in this paper.
